# Assessment of the Service Life of Polyethylene Pipes with Controlled Defects Using Internal Pressure Test

**DOI:** 10.3390/ma18235407

**Published:** 2025-11-30

**Authors:** Ioana-Daniela Manu, Marius Gabriel Petrescu, Cătălin Blag, Ramadan Ibrahim Naim

**Affiliations:** Mechanical Engineering Department, Petroleum-Gas University of Ploiesti, 100680 Ploiesti, Romania; ioana.manu@upg-ploiesti.ro (I.-D.M.); pmarius@upg-ploiesti.ro (M.G.P.)

**Keywords:** polyethylene pipe, controlled geometry pipe, pressure, remaining service life

## Abstract

Controlled geometry defects can be volumetric defects, usually located on the outer surface of the pipe, having different orientations and lengths and identical depths. This type of defect corresponds to the type obtained using a damage mechanism, as presented by API 579-1/ASME FFS-1, Part 9, called crack-type defects. The research presented in this paper was intended to evaluate the influence of controlled defects on the strength of an HDPE water pipe, PE100 (Ø 90 × 5.4), SDR 17, PN 10 bar, subjected to internal pressure. The methods applied were the internal pressure test and numerical simulation. The article’s main findings were the critical pressure P_cr_, the critical time t_cr_, the critical depth of defect a_cr_, and the remaining service life t. The remaining service life was approximately 83 years for the pipe with a defect oriented circumferentially, and 69 years for the pipe with a defect oriented longitudinally.

## 1. Introduction

Researching the influence of defects on the strength of high-density polyethylene (HDPE) pipes is imperative because of the presence of defects, such as lack of material, geometric discontinuities, and cracks, which can result from manufacturing, transportation, storage, and/or installation [1].

The article proposes a method for evaluating the mechanical behavior of defective HDPE water pipes made of PE100 (Ø 90 × 5.4), SDR 17, PN 10 bar, under internal pressure, presenting with defects with controlled geometry that correspond to a level 2 assessment of crack-like flaws, according to Part 9 of the Fitness-For-Service (FFS) regulations [2].

Such defects can initiate braking points when transient flows in these pipes occur during operation—especially when water hammer phenomena occur [3,4,5,6].

HDPE pipes without defects are characterized by a certain lifetime value under normal operating conditions. HDPE pipes have a remaining service life, or lifespan, before they require replacement or repair due to normal damage [7]. Under optimal operating conditions, HDPE pipes work longer than the estimated 50 years, and have a service life of up to 100 years under good monitoring [8].

The fitness-for-service assessment of defective pipelines is a quantitative evaluation of whether the defective pipelines are suitable for continuous use and how to continue to use them [9].

The mechanical performance of a traditional notched HDPE pipe (NHP) with various groove depths and shapes, including U-type, V-type, and L-type (linear type), are critical for the safety assessment [10].

In [9], a numerical analysis was performed in which the defect length was varied, depending on the outer diameter of the pipe, the pipe wall thickness, and the order of magnitude of the defect length. The maximum von Mises stress in the pipeline gradually increased with increasing defect length. When the defect length reached 30 mm, the maximum von Mises stress had almost no effect on the von Mises stress in the pipeline. When the order of magnitude of the axial length of the pipeline was greater than 2, the critical failure load of the pipeline tended to remain unchanged [9].

The scientific purpose of this article is to determine the critical pressure P_cr_, the critical time t_cr_, the critical depth of defect a_cr_, and the remaining service life t for HDPE pipes containing controlled defects, through the internal pressure test and numerical simulation.

## 2. Materials and Methods

### 2.1. Materials

#### 2.1.1. Pipes and End Caps

For testing of polyethylene pipes under the internal pressure test, three sections of HDPE water pipe, PE100 (Ø 90 × 5.4), SDR 17, PN 10 bar were used, 300 mm in length. The sections were cut from a pipe intended for drinking water distribution networks.

The PE100 material has the physical and mechanical parameters indicated in Table 1. All values were recorded at 23 °C.

The pipes were tested safely thanks to the two type A steel end caps [13].

**Figure 1:**
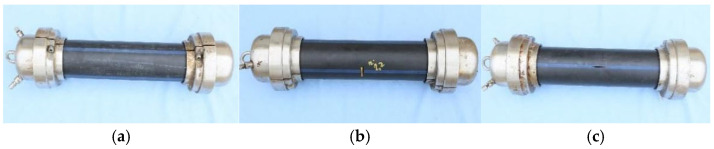
The pipes with and without defects practiced in a controlled manner, subjected to internal pressure: (**a**) the WD pipe; (**b**) the CD pipe; (**c**) the LD pipe.

Three pipe samples were used in the research, as shown in Figure 1: (WD)—pipe without defect (Figure 1a), (CD)—pipe with a defect oriented circumferentially (Figure 1b), and (LD)—pipe with defect oriented longitudinally (Figure 1c).

The dimensions of the defects on the outer surface of the two pipes are presented in Table 2.

#### 2.1.2. Experimental Installation for Internal Pressure Test of HDPE Pipes

For the internal pressure test of the three PE100 pipes, the experimental installation, called IPT Equipment, model 1751-0313, produced by IPT für Prüftechnik Gerätebau GmbH & Co. KG, Todtenweis, Germany, was used, as described in [14].

The pressure machine was calibrated by the manufacturer, and the internal pressure was monitored using a digital pressure transducer with an accuracy of ±0.05 bar (±5 kPa), corresponding to 99.5% accuracy at a pressure of 10 bar. The equipment is periodically maintained and recalibrated according to the manufacturer’s specifications.

#### 2.1.3. Milling Cutter Used for Cutting Notches

The notches made on the outer surface of the pipes, which constitute the controlled defects, were carried out with an angular milling cutter at 45°.

### 2.2. Test Method

#### 2.2.1. Assessment of Crack-like Flows According to Part 9 of Fitness-for-Service (FFS)

In general, the structural integrity of a pipe under internal pressure can be evaluated by a limit state criterion, represented by the equivalent stress σ_ech_.

The expression of the equivalent stress σ_ech_ is obtained by adaptation from [15], according to the Huber–Hencky–Mises version of the energy hypothesis, which considers only the energy from variations in shape.

The adaptation consists of the equivalent principal stresses σ_1_, σ_2_, and σ_3_ with circumferential stress σ_c_, longitudinal stress σ_l_, and radial stress σ_r_, thus σ_1_ = σ_c_, σ_2_ = σ_l_, and σ_3_ = σ_r_.

The equivalent stress is determined by Equation (1).

σ_ech_ = {1/2[(σ_c_ − σ_l_)^2^ + (σ_l_ − σ_r_)^2^ + (σ_r_ − σ_c_)^2^]}^1/2^ (1)

The structural integrity of a thin-walled pipe under internal pressure presenting a defect located on the outer surface can be evaluated by the equivalent stress σ_ech_, which is computed with relationship (2) and adapted for radial stress, σ_r_ = 0.

σ_ech_ = (1/2)∙(σ_c_ − σ_1_)^1/2^, (2)

The circumferential stress σ_c_ is obtained according to [1], depending on the maximum recorded internal pressure P_i_ for bursting in the tested pipe with outer diameter D_e_, wall thickness s, and without defects, with relationship (3).

σ_c_ = [(D_e_ − s)∙P_i_]/(2∙s), (3)

The longitudinal stress value σ_l_ is half of the circumferential stress value σ_c_ [16].

According to [17], the circumferential stress σ_c_ in the case of pipes with an outer surface defect is determined by replacing the difference (D_e_ − s) with mean diameter D_m_ and maximum recorded internal pressure P_i_, with the crack initiation pressure P_crack_, through relationship (4).

σ_c_ = (D_m_∙P_crack_)/(2∙s), (4)

According to [18], the crack initiation pressure P_crack_ is computed with relationship (5), depending on yield strength σ_y_, outer radius R_e_, inner radius R_i_, the depth of the defect a, the wall thickness s, and the half-length of the defect measured in the direction in which the defect extends.

P_crack_ = σ_y_∙{[((R_e_)^2/^(R_i_)^2^) − 1]/[1 + (4∙(R_e_)^2^)/(R_e_ + R_i_)^2^)]∙[1 − ((a/s)/(1 + (0.3/(2c)/(4∙R_e_)))^1/2^]}, (5)

The highest value of the critical pressure P_cr_ is recorded in the case of pipes without defects. The explanation could be that the presence of a defect in the pipe material would cause the stress to concentrate near the geometric defect. This concentration accelerates the pipe’s rupture under the influence of internal pressure.

The remaining service life t of an HDPE pipe operated under optimal pressure and temperature conditions that presents an initial defect of known dimensions can be determined using a calculation formula that considers the stress intensity factor, K [19].

The initial defect of known dimensions can occur in one of three modes of independent kinematic movements, as described by Irvin [20]. One of the three modes can be mode I, or the mode of tensile opening, which is characterized by the stress intensity factor K_I_. The stress intensity factor K_I_ is included in the toughness ratio K_r_. The toughness ratio K_r_, together with the load ratio L_r_, represents the coordinates of the Failure Assessment Diagram (FAD) [21]. The relationships for determining the values of these coordinates are relationship (6) and (7), respectively.

L_r_ = σ_c_/σ_y_, (6)

K_r_ = K_I/_K_Ic_, (7)

The relationship between the stress intensity factor for mode I, K_I_, and the critical stress intensity factor K_Ic_, given by relationship (8), is used to evaluate the non-initiation condition of the rupture process of the HDPE pipe.

K_I_ ≤ K_Ic_, (8)

According to the API 579-1/ASME FFS-1 standard, evaluation level 2 [2], the pairs of points that constitute the curve representing the FAD diagram are obtained by relationship (9) [21].

K_r_ = [1 − 0.14 (L_r_)^2^]∙{0.3 + 0.7∙exp [−0.65 (L_r_)^6^]}, (9)

The HDPE pipe is broken at critical pressure value P_cr_, corresponding to the critical time value t_cr_, and the critical depth of defect, a_cr_.

#### 2.2.2. Internal Pressure Test

For experimental testing, a metallic Bourdon-type pressure gauge was mounted on one of the ends of the tested HDPE pipe. The automatic data recording system was also attached to the same end. The experimental system provides protection against shock through the one-way valve.

At the opposite end, the HDPE pipe was filled by supplying water at 23 ± 2 °C, under pressure.

The slow and progressive increase in pressure during the test was maintained until bursts/failures occurred in the pipes. The pressure from the specimens was continuously recorded through the automatic data recording system IPT Data Logging, model no. 1780, produced by IPT Institut für Prüftechnik Gerätebau GmbH & Co. KG, Todtenweis, Germany.

#### 2.2.3. Failure Assessment Diagram (FAD)

The two coordinate axes of the FAD, namely the X-axis and Y-axis, represent the load ratio L_r_ and toughness ratio K_r_, respectively.

On the coordinate axes, the benchmarks from 0 to 1.4 are considered as an example.

The FAD shows two operating regions: safe and potentially unsafe.

The curve in the FAD is plotted by positioning the pairs of assessment points (L_ri_, K_ri_).

The assessment points (L_ri_, K_ri_) represented under the curve in the FAD indicate the acceptable defects, and the assessment points (L_ri_, K_ri_) represented over the curve in the FAD indicate the unacceptable defects.

#### 2.2.4. Numerical Analysis

To accurately characterize the behavior of HDPE pipes subjected to internal pressure, the numerical analysis using the finite element method FEM was carried out in the ANSYS program.

The influence of the presence of defects on the strength of HDPE pipes was highlighted by increasing the depth and the length of the defects from 10% to 70% of the depth and the length of the respective pipes.

**Figure 2:**
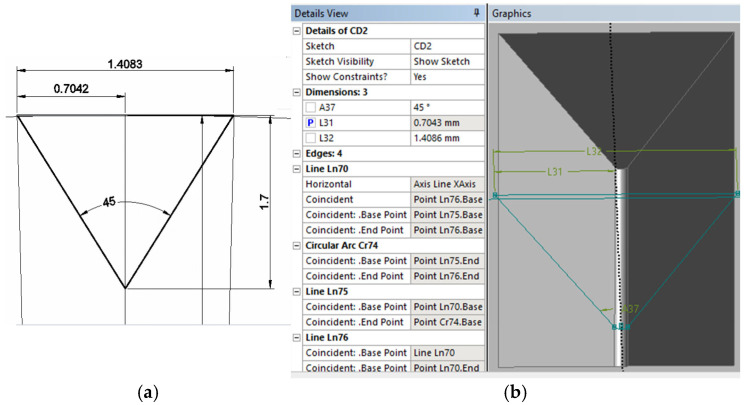
The representation of the defect: (**a**) in AutoCAD program; (**b**) in DesignModeler.

In the GEOMETRY stage of the ANSYS program, the representation of the defect in the plan drawn in the AutoCAD 2024 program, shown in Figure 2a, and the sketch of the defect made in Design Modeler 2024, shown in Figure 2b, were used.

In the numerical analysis the element size used in the meshing step was the default, both in the pipe wall and in the areas where defects were modeled.

For the WD pipe, 5053 discretization nodes connected in 3253 elements in total were used, of which 2461 were Solid 187 and 792 were SURF 154 elements.

#### 2.2.5. Determining the Remaining Service Life

The remaining service life t is one of the factors on which the efficiency of the polyethylene pipeline system used for water distribution depends. The formula for determining the remaining service life, according to [19], is presented in relationship (10), where a_i_ is the initial defect depth.

(10) $$t=\int_{a_{i}}^{s}\frac{1}{5.54\times 10^{-8}\times K_{I}^{4.66}}da,$$

The analytical expression of the stress intensity factor K_I_, according to [22], is presented in relationship (11).

K_I_ = {M_F_ + [Φ∙(c/a)^1/2^ − M_F_]∙(a/s)^8^}∙{[σ∙(π∙a)^1/2^]/Φ}∙M_TM_, (11)

M_F_ is the factor that depends on the geometry of the defect (a/c). This factor is determined by relationship (12).

M_F_ = [1 − (a/c)^2^] ^1/2^, (12)

Φ is the complete elliptic integral of the second degree, which is determined by relationship (13).

(13) $$\Phi =\int_{0}^{\pi /2}\sqrt{1-e_{f}^{2}\times \sin^{2}\varphi }\times d\varphi ,$$

In relationship (13), e_f_ is the elliptical modulus or eccentricity, determined with relationship (14), depending on the defect angle, φ, which is calculated by relationship (15).

e_f_ = tanφ, (14)

φ = cos^−1^ (a/c), (15)

M_TM_ is the correction factor that considers that the increase in stress is due to the radial deformation in the vicinity of the defect, and which is determined by relationship (16).

M_TM_ = {1 − [(a/s)/M_T_]/[1 − (a/s)]}, (16)

M_T_ is the Folias correction factor, for λ < 1, which is determined by relationship (17).

M_T_ = (1 + 1.61∙λ^2^)^1/2^, (17)

λ is a ratio that is determined, depending on the mean radius, R_m_, by relationship (18).

λ = c/(R_m_∙s)^1/2^, (18)

The mean radius, R_m_, represents the arithmetic mean between the outer radius, R_e_, and the inner radius, R_i_. The mean radius, R_m_, is determined by relationship (19).

R_m_ = (R_e_ + R_i_)/2, (19)

**Figure 3:**
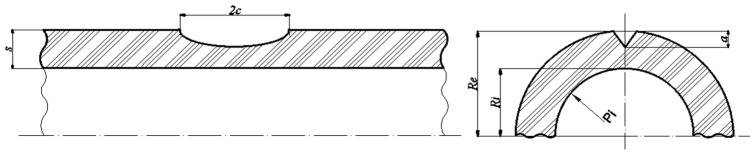
Section in PE100 pipe showing an unpenetrated semi-elliptic defect on the outer surface [1].

To highlight the geometric characteristics of the PE100 pipe and the unpenetrated semi-elliptic defect positioned on its outer surface, wall thickness s, outer and inner radii R_e_ and R_i_, defect length 2c, and defect depth a were presented in Figure 3. The internal pressure P_i_ was also noted in Figure 3.

## 3. Results

### 3.1. Results Obtained from the Internal Pressure Test

#### 3.1.1. Bursting Failure Analysis

**Figure 4:**
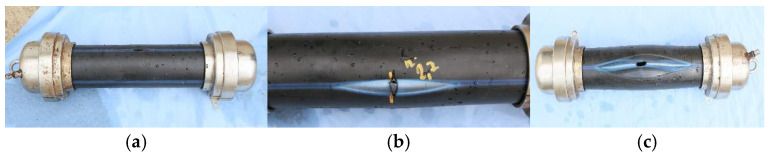
Pipes without and with controlled defects, which burst in the internal pressure test: (**a**) pipe without defect; (**b**) CD pipe; (**c**) LD pipe.

Pipes without and with controlled defects, which burst in the internal pressure test, are presented in Figure 4.

The burst in the pipe without a defect was a brittle fracture that occurred without significant pipe deformation. The failure was manifested by the development of very fine cracks, which had a parallel direction relative to the pipe axis.

The failure of the pipe with a circumferentially oriented defect was a ductile failure. The pipe material was strongly deformed and expelled outwards.

The failure of the pipe with a longitudinally oriented defect was parrot-beak type, and occurred when the pipe swelled and the wall thinned until failure. The crack had the same direction as the pipe axis.

#### 3.1.2. Values of Critical Pressure P_cr_, and Critical Time t_cr_

The values of critical pressure P_cr_ and critical time t_cr_ recorded for the three PE100 pipes subjected to the internal pressure test are presented in Table 3.

A simple statistical analysis of the critical pressure and time values indicates consistent differences between the pipe’s conditions, reinforcing the reliability of the experimental observations.

The role of the experimental test data is to discover and demonstrate that a circumferentially oriented defect will lead to a slower failure of the pipe than if the defect is oriented longitudinally (in which case the principal stress is perpendicular to the defect contour in the direction of the major axis).

### 3.2. Results Obtained When Drawing the FAD

The input data required to determine the two pairs of assessment points are shown in Table 4. The values of circumferential stress σ_c_ for both tested pipes (the CD pipe and the LD pipe) were computed with relationships (4) and (5). The value of yield strength σ_y_ was mentioned in Table 1. The values of load ratio L_r_ were calculated with relationship (6). The values of stress intensity factor K_I_ were calculated with relationship (11). The value of critical stress intensity factor K_Ic_ was mentioned in Table 1.

### 3.3. Results Obtained Through Numerical Analysis

**Figure 5:**
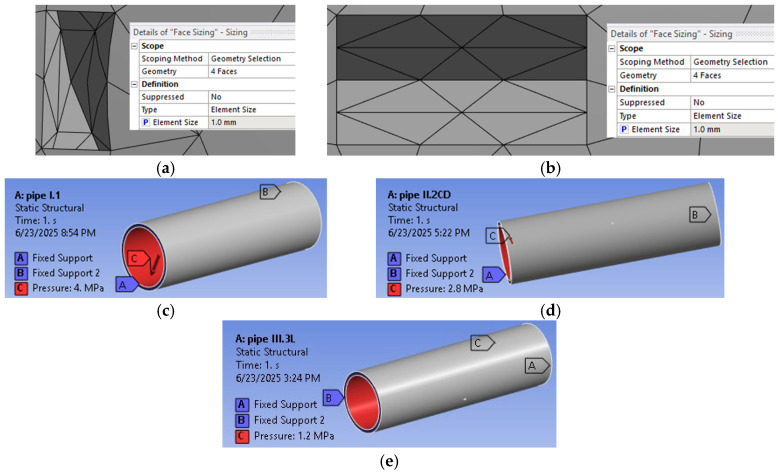
The details of the dividing grids (**a**,**b**) and the boundary settings (**c**–**e**).

The details of the dividing grids are presented in Figure 5a,b. The boundary settings are presented in Figure 5c–e.

**Figure 6:**
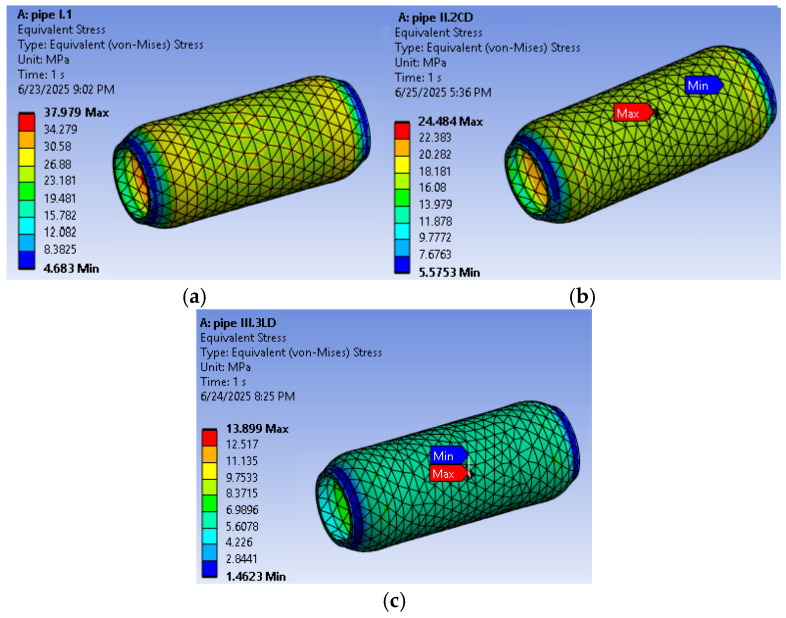
The results of the numerical evaluation under equivalent (von Mises) stress: (**a**) WD pipe; (**b**) CD pipe; (**c**); LD pipe.

The results of the numerical evaluation of pipes with and without controlled defects, as well as the equivalent stress distribution, are presented in Figure 6a–c.

### 3.4. Results About Remaining Life

This evaluation applies to pipes with standard dimensional ratios (SDR) of 7.4, 6, and 5, and pipes that withstand permissible operating pressure between 24.4 and 10 bar after 10, 25, and 50 years of operation [23].

The influence of the defect depth on the equivalent stress that develops in the pipe wall is presented in relationship (20) for the CD pipe, and in relationship (21) for the LD pipe.

The influence of the defect length of the equipment stress that develops in the pipe wall is presented in relationship (22) for the CD pipe, and in relationship (23) for the LD pipe.

σ_ech,a,CD_ = 2.6667a^2^ − 5.5808a + 28.785 (20)

σ_ech,a,LD_ = −0.0016a^3^ + 0.0125a^2^ − 0.03x + 10.986 (21)

σ_ech,2c,CD_ = 0.0009(2c)^4^ − 0.0385(2c)^3^ + 0.57(2c)^2^ − 2.7514(2c) + 30.682 (22)

σ_ech,2c,LD_ = −2E−06(2c)^4^ + 0.0001(2c)^3^ − 0.0017(2c)^2^ + 0.0109(2c) + 10.944 (23)

## 4. Discussion

### 4.1. Discussion About Internal Pressure Test

For the comparative study of the behavior of the three pipes subjected to the internal pressure test, the values of the internal pressure, the circumferential stress, and the longitudinal stress are presented in Table 5.

### 4.2. Discussion About Numerical Analysis

The values of equivalent stress for the pipes with circumferentially and longitudinally oriented defects under the conditions of changing the defect depth are presented in Table 6.

The increase in defect depth causes an insignificant increase in equivalent stress in the pipe wall.

The values of equivalent stress for the pipes with circumferentially and longitudinally oriented defects under the conditions of changing the defect length are presented in Table 7.

The increase in defect length causes a significant increase in equivalent stress in the pipe wall when the defect length reached 30 mm. This confirms the conclusion from [9], for the pipe with a defect oriented longitudinally.

**Figure 7:**
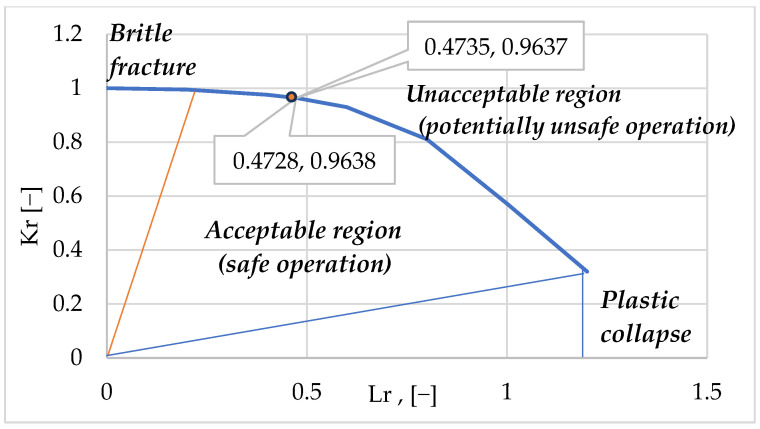
The FAD (processed after [21]).

For the pipe with the circumferentially oriented defect, the critical depth of the defect a_cr_ was calculated by substituting the equivalent stress–defect depth relationship, as shown in Figure 7. This dependency relationship was rewritten by replacing the term y with the maximum equivalent stress corresponding to the pipe without defects, σ_ech_ = 37.976 MPa, and the term x with the critical depth of defect a_cr_, obtaining Equation (24).

2.6667∙(a_cr_)^2^ − 5.5808∙(a_cr_) + 28.785 = 37.976, (24)

The determined critical depth of defect value is a_cr_ = 3.1775 mm.

### 4.3. Discussion About FAD

In the FAD, represented in Figure 7, the curve that contains the two pairs of points (L_r_, K_r_) is plotted (0.4728, 0.9638) and (0.4735, 0.9637), respectively, corresponding to the two defects on the outer surface of the defective pipes.

In the FAD, the curve separates the two operating regions, the acceptable region (safe operation) and the unacceptable region (potentially unsafe operation). The thin red line represents the boundary of the brittle region, and the blue line shows the plastic collapse boundary. Both regions are predicted by the FAD.

### 4.4. Discussion About the Remaining Service Life

To determine the remaining service life of pipes with controlled defects subjected to the internal pressure test, relationships (10)–(18) were used. The input data used to determine the values of the remaining service life are shown in Table 8. The results are presented in Equations (25) and (26). To accurately determine the remaining service life of pipes with controlled defects, the number 8760 h/year was considered.

(25) $$t=\int_{0.0017}^{0.0054}\frac{1}{5.54\times 10^{-8}\times 0.5986^{4.66}}dx=729852$$

(26) $$t=\int_{0.0017}^{0.0054}\frac{1}{5.54\times 10^{-8}\times 0.6224^{4.66}}dx=608594$$

Compared to similar studies [7,8], the results show compatible trends in the influence of defect geometry on failure pressure and lifetime prediction, confirming the validity of the assessment methodology applied in this work.

### 4.5. Comparison Between the Results of the Experimental Test and Numerical Evaluation

**Figure 8:**
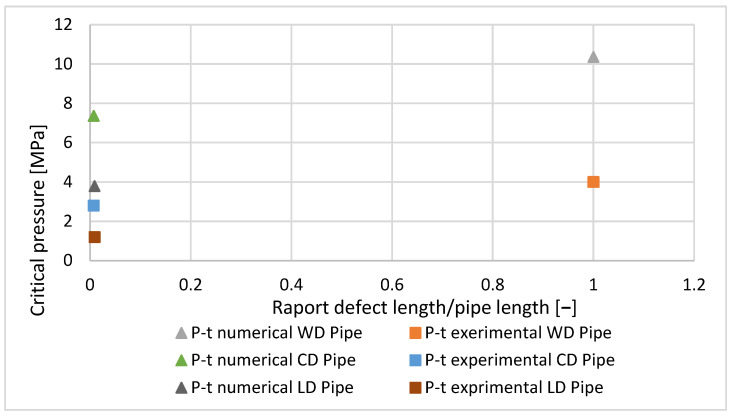
Comparison between the results of the numerical simulation and the results of the experimental test.

The numerical evaluation had the same conditions (pressure and defect dimensions) as the internal pressure test. For this reason, the results of numerical simulation and the results of the internal pressure test were compared to find out where they agreed. The comparison is illustrated in Figure 8.

The maximum critical pressure values correspond to the pipe without defects, and are as follows: 10.37 MPa for the numerical simulation and 4 MPa for the experimental test.

In Figure 8 there is no agreement between the experimental and numerical results because the linear elastic constitutive model of the material was used in the FEM, although the material has nonlinear viscoelastic properties. For this reason, the maximum stress of 37.979 MPa is higher than the yield stress of the material.

## 5. Conclusions

This study provides a comprehensive framework for evaluating the service life of HDPE pipes with outer surface defects of controlled geometry, by integrating an experimental internal pressure test, numerical simulation through finite element analysis, and fracture mechanism via the FAD.

This approach departs from generalized or empirical assessment by enabling defect-specific predictions of burst pressure, critical time to failure, and remaining service life based on defect type, orientation, and dimensions.

According to the experimental test data, a circumferentially oriented defect will lead to a slower failure of the pipe than a longitudinally oriented defect (where the principal stresses are perpendicular to the defect contour in the direction of the major axis).

Through the application of the FAD methodology, supplemented with finite element simulation, the study demonstrates that both safe and unsafe defect conditions can be discriminated with high reliability. The possible correlation between experimental and numerical results further validates the applicability of the proposed methodology.

These findings have direct implications for pipeline design, condition assessment, and maintenance strategies. By quantifying the influence of the defect orientation and geometry, the results support optimized inspection intervals and risk-informed replacement decisions for water distribution systems employing HDPE infrastructure.

Moreover, the methodology developed is transferable to other pressurized thermoplastic pipeline systems, such as those used in gas distribution or industrial fluid transport, offering a scalable tool for structural integrity assessment under real-world operating conditions.

A notable limitation of this study is the reduced length of the tested pipe segments, which may not fully replicate the stress distributions encountered in long-span networks. Future research may address this by extending the analysis to full-scale pipes, including internal or multiple defect scenarios, and incorporating variable environmental factors such as temperature fluctuations or external mechanical loads.

Overall, this research contributes a validated, physics-based approach for evaluating the mechanical resilience and residual life of HDPE pipelines with outer surface flaws, reinforcing the role of fracture mechanics in predictive maintenance and infrastructure safety.

## Figures and Tables

**Table 1:** The PE100 type of HDPE material has the following physical and mechanical parameters.

Physical and Mechanical Characteristics Name and Symbol	Values and Units	Methods
Density, ρ	958–960 [g/cm^3^]	ISO 1183 [11]
Poisson ratio, ν	0.45 [−]	Experimental testing ^1^
Young’s modulus, E	1100 [MPa]	ISO 527 [12]
Yield strength, σ_y_	29.6234 [MPa]	Experimental testing
Ultimate tensile strength, σ_t_	14.6082 [MPa]	Experimental testing
Tensile elongation, ε_r_	737.7127 [%]	Experimental testing
Critical stress intensity factor, K_Ic_	0.742 [MPa m^1/2^]	Experimental testing

^1^ Experimental Testing was carried out on PE100 specimens [1].

**Table 2:** The width, length, and depth of defects practiced in a controlled manner in two pipes.

Tested Pipe	Defect Width, l, [mm]	Defect Length, 2c, [mm]	Defect Depth, a, [mm]
CD pipe ^1^	1.41	2.2	1.7
LD pipe ^2^	1.41	2.7	1.7

^1^ CD—pipe with a defect oriented circumferentially; ^2^ LD—pipe with a defect oriented longitudinally.

**Table 3:** The values of critical pressure P_cr_ and critical time t_cr_ were recorded for the three PE100 pipes.

Tested Pipe	Critical Pressure P_cr_, [MPa]	Critical Time t_cr_, [s]
WD pipe	4.0	58
CD pipe	2.8	29
LD pipe	1.2	28

**Table 4:** The input data required to draw the FAD are presented under the mechanical parameters.

Tested Pipe	Circumferential Stress σ_c_, [MPa]	Yield Strength σ_y_, [MPa]	Load Ratio L_r_, [−]	Stress Intensity Factor K_I_, [MPa∙m^1/2^]	Critical Stress Intensity Factor K_Ic_, [MPa∙m^1/2^]	Toughness Ratio K_r_, [−]
CD pipe	14.0257	29.6234	0.4735	0.6224	0.743	0.8377
LD pipe	14.0061		0.4728	0.5986		0.8057

**Table 5:** The values of internal pressure, the circumferential stress, and the longitudinal stress are presented for the comparative study of behavior of the three pipes subjected to the internal pressure test.

Tested Pipe	Internal Pressure P_i_, [MPa]	Circumferential Stress σ_c_, [MPa]	Longitudinal Stress σ_l_, [MPa]
WD pipe	4.0	31.333	15.6667
CD pipe	2.8	21.933	10.9667
LD pipe	1.2	9.4	4.7

**Table 6:** The values of equivalent stress for the pipes with circumferentially and longitudinally oriented defects change with the change in defect depth.

Defect Depth a, [mm]	CD Pipe	LD Pipe
	Equivalent Stress σ_ech_, [MPa]	
0.54	23.126	12.469
1.08	22.562	15.98
1.62	23.365	14.108
**1.7**	**24.484**	**13.899**
2.16	22.475	13.785
2.7	23.967	12.368
3.24	24.103	13.408
3.78	23.293	12.942

**Table 7:** The values of equivalent stress for the pipes with circumferentially and longitudinally oriented defects change with the change in defect length.

CD Pipe		LD Pipe	
Length Defect 2c, [mm]	Equivalent Stress σ_ech_, [MPa]	Length Defect 2c, [mm]	Equivalent Stress σ_ech_, [MPa]
**2.2**	**24.484**	**2.7**	**13.899**
30	23.241	30	19.221
60	23.431	60	19.608
90	23.562	90	18.537
120	24.231	120	19.723
150	23.679	150	20.416
180	23.703	180	21.14
210	23.474	210	18.997

**Table 8:** The values of the remaining service life of pipes with controlled defects subjected to the internal pressure test are computed as functions of the geometrical and mechanical characteristics.

**Tested Pipe**	**Outer** **Diameter** **D_e_, [m]**	**Defect Depth** **a, [m]**	**Defect Length 2c, [m]**	**Defect Half-Length** **c, [m]**	**Mean** **Radius** **R_m_, [m]**	**Wall Thickness s, [m]**	**Ratio** **λ, [−]**	**Folias** **Correction Factor** **M_T_, [−]**
CD pipe	0.09	0.0017	0.0022	0.0011	0.0423	0.0054	0.0738	1.0044
LD pipe			0.0027	0.0135				
**Tested pipe**	**Correction** **factor M_TM_, [−]**	**Defect angle φ, [°]**	**Elliptic modulus or eccentricity e_f_, [−]**	**Complete** **elliptic integral of second degree** **Φ, [−]**	**Factor depends on the defect** **geometry** **M_F_, [−]**	**Equivalent** **stress** **σ_ech_, [MPa]**	**Stress intensity** **factor** **K_I_,** **[MPa∙m^1/2^]**	**Remaining service life** **t, [years]**
CD pipe	1.0009	3.94	1.0264	0.7549	0.953	13.0046	0.5986	83.3164
LD pipe		3.26	0.1214	1.4755		13.1739	0.6224	69.4742

## Data Availability

The original contributions presented in this study are included in the article. Further inquiries can be directed to the corresponding authors.

## Abbreviations

The following abbreviations are used in this manuscript:

API	American Petroleum Institute
ASME	American Society of Mechanical Engineers
FAD	Failure assessment diagram
FEM	Finite element method
FFS	Fitness-for-service
HDPE	High-density polyethylene
NHP	Traditional notched HDPE pipe
PE100	High-density polyethylene material with a minimum required strength of 10 MPa
PN	Nominal pressure
CD	Pipe with a defect oriented circumferentially
LD	Pipe with a defect oriented longitudinally
WD	Pipe without defect
SDR	Standard dimensional ratio
a	Defect depth [mm]
a_i_	Initial defect depth [mm]
a_cr_	Critical depth of defect [mm]
2c	Defect length, measured in the direction in which the defect extends [mm]
D_e_	Outer diameter of pipe [mm]
σ_c_	Circumferential stress [MPa]
σ_ech_	Equivalent stress [MPa]
σ_l_	Longitudinal stress [MPa]
σ_r_	Radial stress [MPa]
e_f_	Elliptic modulus or eccentricity [−]
E	Young’s modulus [MPa]
K	Stress intensity factor [MPa∙m^1/2^]
K_I_	Stress intensity factor for mode I of opening through tensile [MPa∙m^1/2^]
K_Ic_	Critical stress intensity factor [MPa∙m^1/2^]
K_r_	Toughness ratio [−]
L_r_	Load ratio [−]
M_F_	Factor that depends on the geometry of the defect (a/c) [−]
M_T_	Folias correction factor [−]
M_TM_	Correction factor that considers the increase in stress due to the radial deformation [−]
P_i_	Maximum recorded internal pressure [MPa]
P_cr_	Critical pressure [MPa]
P_crack_	Crack initiation pressure [MPa]
R_e_	Outer radius [mm]
R_i_	Inner radius [mm]
R_m_	Mean radius [mm]
s	Wall thickness [mm]
t	Remaining service life [years]
t_cr_	Critical time [s]
ε_r_	Tensile elongation at 23 °C
λ	Ratio determined by mean radius [−]
ν	Poisson ratio [−]
ρ	Density at 23 °C
σ_c_	Circumferential stress [MPa]
σ_ech_	Equivalent stress [MPa]
σ_l_	Longitudinal stress [MPa]
σ_r_	Radial stress [MPa]
σ_tr_	Ultimate tensile strength at 23 °C [MPa]
σ_y_	Yield strength [MPa]
